# Repetitive transcranial magnetic stimulation enhances alpha power in Alzheimer's disease patients

**DOI:** 10.1177/13872877251406972

**Published:** 2025-12-29

**Authors:** Ronja Verena Fassbender, Christina Kehm, Anna-Lisa Otta, Gereon Rudolf Fink, Oezguer Abdullah Onur

**Affiliations:** 1 Cognitive Neuroscience, Institute of Neuroscience and Medicine (INM-3), Research Center Jülich, Jülich, Germany; 2 Department of Neurology, Faculty of Medicine and University Hospital Cologne, University of Cologne, Cologne, Germany

**Keywords:** alpha rhythm, Alzheimer's disease, EEG, episodic memory, noninvasive brain stimulation, repetitive transcranial magnetic stimulation

## Abstract

**Background:**

With Alzheimer's disease (AD) presenting an ongoing challenge, innovative treatment methods are essential. Repetitive transcranial magnetic stimulation (rTMS) has emerged as a promising noninvasive intervention, particularly targeting alpha band oscillations associated with AD-related cognitive decline.

**Objective:**

This study aimed to investigate the effects of low-intensity rTMS over posterior cortical areas on alpha band oscillations and memory performance in AD patients compared to age-matched healthy controls.

**Methods:**

In a single-blinded, sham-controlled rTMS-EEG study, we examined 14 amyloid-positive AD patients and 14 age-matched healthy controls. Continuous EEG was recorded at rest (eyes closed) before, during, and after stimulation. During stimulation, participants completed an episodic memory task.

**Results:**

We were able to demonstrate that during rTMS alpha power increased compared to sham, with a notable 25% increase observed in AD patients. However, comparison of memory performance under the sham and stimulation conditions revealed no significant stimulation effect.

**Conclusions:**

These findings support and extend current knowledge of noninvasive brain stimulation mechanisms. Our results suggest that alpha frequency-tuned rTMS over posterior cortical areas can modulate pathological brain activity in AD patients even at low intensities. Given the limited sample size and moderate effect sizes, results should be interpreted with caution. Nevertheless, our results warrant further studies with long-term EEG-rTMS protocols to evaluate the potential therapeutic benefit.

## Introduction

As life expectancy is constantly growing, the number of people suffering from dementia and the associated social and economic consequences will further increase. Alzheimer's disease (AD) is the main cause of dementia.^1^ So far, the efficacy of drugs approved for AD on memory decline, as the leading symptom of the disease, has been disappointing. Recently, lecanemab, an anti-amyloid agent, was approved by the FDA and gives reason for hope, even though the data of its predecessor, aducanumab, were not entirely convincing.^2^ Experts on pharmacotherapy consider combination therapies as particularly promising.^3^ Therefore, non-pharmacological therapies for patients with AD are urgently needed, regardless of the success of pharmacological treatments. A promising therapeutic approach, which is already approved for treating neuropsychiatric disorders such as depression, is non-invasive brain stimulation (NIBS).^4^ Repetitive transcranial magnetic stimulation (rTMS) is a NIBS technique based on the principle of electromagnetic induction.^5^ Through rTMS neuronal excitability is modulated and neuronal plasticity is induced.^4^ For clinical use, the stimulation protocol needs to be adapted to the disease being treated in terms of stimulation site, frequency, and intensity.

In most of the studies on AD patients, the dorsolateral prefrontal cortex (DLPFC) was stimulated by high-frequency rTMS (>10 Hz), yielding positive effects mainly on various language functions and global cognition.^6–8^ In a few more recent studies on AD patients, posterior regions including the precuneus were stimulated showing positive effects on memory, whose impairment represents the leading symptom of the disease.^9–11^ For example, Koch et al.^12^ demonstrated that long-term rTMS over the precuneus preserved cognitive function and activities of daily living over 52 weeks. Positron emission tomography (PET) or functional magnetic resonance imaging (fMRI) studies also reveal a central engagement of the precuneus in memory processes, especially in the consolidation and retrieval of episodic memory.^13^ Moreover, the major pathological features of AD, extracellular deposition of amyloid-β (Aβ) plaques and intracellular deposition of neurofibrillary tangles, first affect posterior cortical brain regions, including the precuneus (PC), posterior cingulate cortex, and posterior parietal cortex (PPC).^14,15^ Structural and functional abnormalities in these regions are already observed at early stages of the disease. The results of combined PET-MRI studies suggest that tau pathology within posterior cortical regions may be linked temporally more closely to the observed hypometabolism than to the structural atrophy in this region. Thus, the functional abnormalities are thought to precede the structural abnormalities.^15^ Stimulation of posterior brain regions could therefore foster a positive influence on the metabolism in these regions and influence disease progression by delaying structural atrophy.

Regarding stimulation frequency, studies have provided evidence that the entrainment of natural brain oscillations using NIBS, a technique called rhythmic TMS, a subtype of repetitive TMS (rTMS), appears to be particularly effective.^16^ According to Zheng et al.,^17^ this also applies to patients with amnestic mild cognitive impairment (MCI), as rhythmic TMS modulating the theta band (intermittent theta burst stimulation, iTBS) proved to be more effective than classic rTMS protocols in terms of modulating the temporoparietal network and the associated memory improvement.^18–20^

AD is characterized by a slowing of brain oscillations, which is already observed in the preclinical stages of the disease and may predict clinical progression.^18^ The analysis of the power spectral density shows an increase in slower frequency bands like the theta frequency band (Hz) and a decrease in faster frequency bands like the alpha frequency band, particularly in parietal, temporal, and occipital areas.^19^ The abnormalities in the cortical EEG rhythms of amnestic MCI and AD patients are closely related to gray matter atrophy and cognition.^20^ Especially, alpha oscillations are associated with effective memory encoding,^21^ retrieval,^22^ and consolidation.^23^ Roh et al.^24^ even demonstrated a correlation of retrieval performance in a verbal and a visuospatial memory task with parieto-occipital alpha power in AD patients at different stages of the disease. Consequently, it can be assumed that an increase in alpha power induced by NIBS leads to an improvement in memory performance. Therefore, stimulation at “natural” frequencies (rhythmic TMS) seems promising for therapeutic use in AD, as endogenous brain oscillations are altered in patients and these disease-related changes correlate with the cognitive decline.^25–27^

A number of studies have already shown that rTMS with bursts tuned to the alpha frequency^28,29^ or transcranial alternating current stimulation (tACS) with a sinusoidal alternating current at alpha frequency^30,31^ modulates the respective band. Hamidi et al.^32^ demonstrated that an increase in alpha power elicited by rTMS at mean alpha frequency above parietal areas was associated with improved memory performance. However, these results refer to young, healthy participants and, to our knowledge, have not yet been replicated for AD patients. In a study by Jia et al. (2021), AD patients received 2-week treatment of 10 Hz rTMS over parietal areas. After the treatment episodic memory performance improved. However, as no EEG or fMRI measurements were obtained either simultaneously or after stimulation treatment, it remains unclear whether the stimulation modulated the alpha frequency band and whether this explains cognitive improvement.^33^

By stimulating at natural frequencies, we can presumably take advantage of the brain's natural resonance and therefore much lower field strengths than conventional rTMS may already produce therapeutic effects.^34,35^ Gonzalez-Rosa et al.^36^ even demonstrated effects on the alpha frequency band by stimulating with a field strength hundreds of times lower than conventional TMS. Lower field strengths seem beneficial in terms of safety and portability, allowing treatment to be delivered by patients and caregivers at home. A number of studies show that NIBS has a sustained therapeutic effect only when applied regularly over several weeks.^10,12,37^ Therefore, treatment at home would provide considerable advantages.

The objective of this study was to examine the potential therapeutic effects of low-intensity alpha-rTMS above parieto-occipital brain areas. Based on the literature described above, we expected a stimulation-induced increase in alpha power and an associated improved performance in episodic memory in AD patients and age-matched healthy controls.

## Methods

### Sample

A total of 36 participants were recruited. The study was approved by the Ethics Committee of the University of Cologne (No. 18-336) and conducted in accordance with the Declaration of Helsinki. Patients in the AD group (*N* = 17) were recruited via the memory clinic of the University Hospital of Cologne from June 2019 to August 2020. Inclusion criteria were in accordance with the diagnostic criteria of the NIA-AA Research Framework from 2018^38^: (1) amyloid status was confirmed by cerebrospinal fluid (CSF) Aβ_42_, Aβ_42_/Aβ_40_ ratio, or amyloid PET (Table 1), (2) aggregated tau was assessed by CSF p-Tau or tau PET, and (3) neurodegenerative changes were detected by structural changes in an MRI scan or by the presence of a typical hypometabolism pattern in an FDG-PET scan. Clinically, patients in the AD group met the criteria for MCI to mild dementia. Patients showed objective cognitive deficits (>1.5 *SD* below the mean of the normative sample) in the memory domain and at least one other cognitive domain. Age-matched healthy controls (*N* = 19) were recruited via the Cologne Alzheimer's Prevention Register, which addresses healthy citizens. Based on a neuropsychological examination, participants were evaluated as cognitively normal by an experienced neuropsychologist (<1.5 *SD* below the mean of the normative sample). Exclusion criteria for both groups included the presence of psychiatric diseases, (other) neurodegenerative or neuroinflammatory diseases, and epilepsy. Due to technical issues, protocol deviations concerning the experimental procedure, and failure to meet the inclusion criteria, a total of 8 participants were excluded from further analyses, and 28 participants remained (*N_AD_* = 14, *N_HC_* = 14).

**Table 1.** AT(N) profile of AD participants.

Subject-ID	Aβ_42_ (pg/mL)	Aβ_40_ (pg/mL)	Aβ_42_/Aβ_40_	t-Tau (pg/mL)	p-Tau (pg/mL)	ATN Profile
P01	529.00	9410.00	0.06	660.80	78.00	A + T + N+
P02	NA^a^			NA	NA	A + T? N+
P03	286.1	5006	0.05	700.6	117.1	A + T + N+
P04	NA^c^			NA^c^	NA	A + T? N+
P05	527^b^			335 b	NA	A + T? N+
P06	264^b^			730 b	98^b^	A + T + N+
P07	497.1	10614	0.05	1171.1	370	A + T + N+
P08	715.3	14912	0.05	511.4	174	A + T + N+
P09	566.3	8163	0.07	219.1	67	A + T + N+
P10	517.6	8182	0.06	562.1	83	A + T + N+
P11	248.5	4905	0.051	468.4	69	A + T + N+
P12	680.4	9489	0.07	544.7	67	A + T + N+
P13	NA^c^			NA^c^	NA^c^	A + T + N+
P14	611.7	8093	0.076	671.6	133.00	A + T + N+

Unless otherwise stated, the following threshold values apply: Aβ_42_ > 650, Aβ_42_/Aβ_40_ > 0.1, Tau < 452, p-Tau <61.

^a^ Amyloid-PET (318 MBq F18-Florbetapir).

^b^ Lab results were taken from the medical documentation of another clinic; no threshold values given, but described as AD-compliant.

^c^ Medical documentation only provided information on amyloid and tau status; no exact values were reported.

### Neuropsychological testing

Each participant underwent a comprehensive neuropsychological examination. The assessment included the CERAD test battery^39^ with tests measuring verbal (word list learning, recall, recognition) and visual memory (figures recall), semantic (animals) and phonematic word fluency (S-words;,^40^ confrontational naming (Boston Naming Test), visuoconstruction (figures drawing), selective (TMT-A) and divided attention (TMT-B).^41^ Moreover, the digit span from the Wechsler Memory Scale^42^ was assessed for short-term and working memory and the Letter Digit Substitution Test^43^ was used to assess psychomotor speed. To ensure that no undiagnosed depressive disorder was present, the participants completed the Geriatric Depression Scale.^44^ In addition, AD patients were evaluated regarding their independence in activities of daily living using the Functional Activities Questionnaire.^45^

### Experimental design

**Figure 1.**
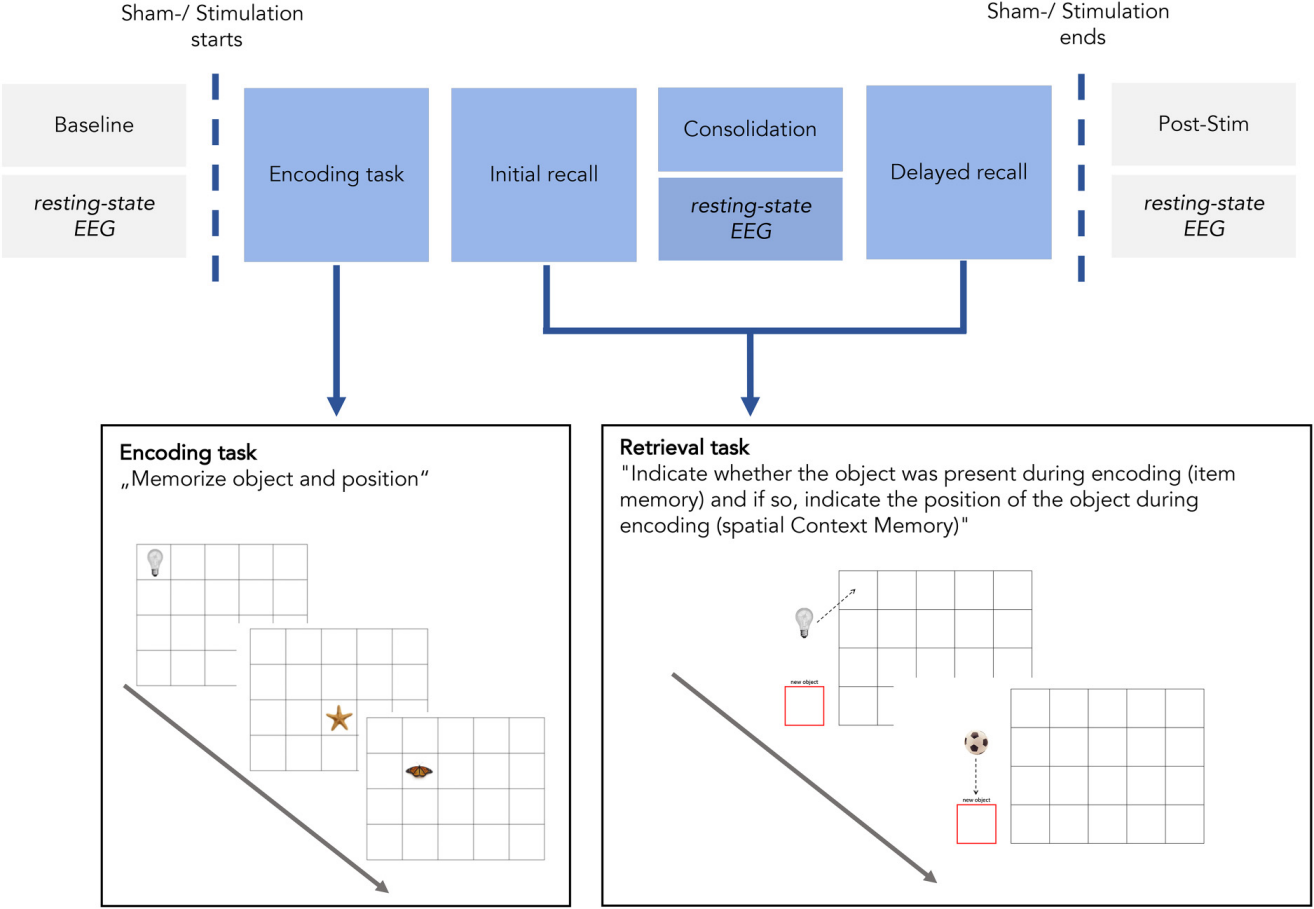
Experimental Design of rTMS-EEG sessions and schematic illustration of the spatial contextual memory paradigm.

The study consisted of three appointments, an initial appointment for the neuropsychological examination and two additional appointments for the non-invasive brain and sham stimulation. There was an interval of approximately 7 days between the appointments (M = 6.48, SD = 3.59). NIBS with high-frequency low-intensity rTMS or sham stimulation, which served as a control condition, was performed simultaneously with EEG measurements (rTMS-EEG). To examine the effect of stimulation on memory performance, a spatial contextual memory task was implemented during the rTMS-EEG sessions. For an overview see Figure 1.

The order of sham and active stimulation conditions was determined by simple randomization prior to data collection. Assignments were applied consecutively as participants were enrolled. Subjects were not informed in which of the two appointments the stimulation occurred. From the participants’ perspective, the procedures during the sham and active stimulation condition were identical. The stimulation device used in this study generates only very low-intensity magnetic fields and therefore does not produce the typical clicking sound or mechanical vibration associated with conventional TMS. After the experiment, the participants answered a questionnaire measuring the sensory impressions during the (sham) stimulation. The results showed that participants could not tell whether they were stimulated or not, suggesting that a placebo effect of stimulation seems unlikely and participants stayed blinded throughout the experiment. Only the experimenter was aware of whether a given appointment corresponded to the sham or active condition, and accordingly either activated or deactivated the stimulation device, resulting in a single-blinded sham-controlled trial.

### Memory paradigm

The memory paradigm consisted of an encoding phase, an initial retrieval, a consolidation phase, and a delayed retrieval. Prior to the experiment, a test run was conducted to familiarize participants with the task. The memory task was a modification of a well-established spatial context memory task.^46–48^ Two sets of stimuli were used, which were counterbalanced across both conditions.

During encoding, ten stimuli were sequentially presented to the participants at one of twenty possible positions in a four-by-five grid on a screen. The ten stimuli were repeated three times in the same position. Participants were instructed to memorize both the stimuli (item memory) and their associated position (spatial context). The stimuli were photographs of either natural (e.g., butterfly) or artificial/man-made (e.g., light bulb) objects. Every object was presented for four seconds. To ensure participants stayed focused on the task, they had to press a button whenever a stimulus appeared.

During an initial retrieval task immediately following the encoding task and during a delayed recall following a ten-minute consolidation period, twenty stimuli (ten previously learned and ten new stimuli) were sequentially presented to the participants in randomized order at a neutral position outside the grid. Participants were instructed to indicate whether the respective stimulus was recognized as previously learned or not (item memory). Stimuli were classified as ‘previously learned’ whenever an object was positioned within the grid irrespective of the exact position. Stimuli were classified as ‘new’ once an object was dragged into a visually highlighted box outside the grid. For stimuli identified as previously learned, participants were further instructed to indicate the object's exact position during encoding by dragging the object to the respective field in the grid (spatial context memory) using a joystick. Participants had ten seconds to respond before the next stimulus appeared.

### High-frequency low-intensity rTMS

**Figure 2.**
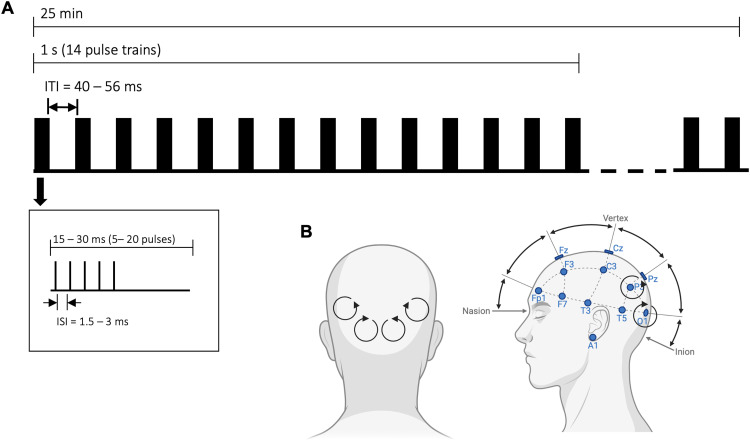
(A) Schematic illustration of high-frequency low-intensity rTMS during the experiment. To improve treatment response, stimulation parameters were individually adjusted, varying the number of pulses per train and the inter-stimulus interval (ISI) within each train. ITI: inter train interval. (B) Schematic illustration of stimulation site.

High-frequency, low-intensity rTMS (<10 mT) was applied using four coils positioned over parieto-occipital brain areas—two over parietal sites and two over occipital sites (see Figure 2). The four coils were embedded into a foam block allowing participants to rest their heads against it while seated upright, thereby stimulating the aforementioned brain areas. It should be noted that precise coil placement was neither feasible nor intended with this device, as neuronavigation was not employed. Electrical pulses were delivered to the magnetic coils in trains repeated with 14 Hz. Several studies indicate that the stimulation effect is strongest when the stimulation frequency is at or near the natural frequency (eigenfrequency) of the brain region being stimulated.^29,49^ Since posterior cortical regions are considered to be the generator of alpha oscillations,^50^ stimulation close to alpha frequency seems reasonable. However, to ultimately distinguish artificial signals of stimulation frequency from endogenous brain oscillations, stimulation should not occur precisely in the frequency range of interest. For this reason, we decided to use a stimulation frequency of 14 Hz in the present study, which corresponds to the upper bound of the alpha band plus 1 Hz.

To optimize each participant's response to the treatment, stimulation parameters were individually adjusted. We first obtained a baseline measurement prior to stimulation. Subsequently, three different stimulation patterns were tested, all consisting of bursts presented at 14 Hz but differing in their internal composition: (a) 5 pulses (15 ms), (b) 10 pulses (30 ms), and (c) 20 pulses (30 ms). Each participant received all three stimulation patterns for two minutes each, presented in randomized order. Quantitative EEG (qEEG) was recorded during stimulation, and the data were analyzed to identify which pattern induced the strongest increase in alpha power. The stimulation pattern that produced the maximal alpha modulation relative to the others was then selected for the main experiment, during which stimulation was applied for a total of 25 min in parallel with the memory paradigm (see Figure 2). A chi-square test showed that the stimulation patterns were evenly distributed across participants (pattern a: n = 15; pattern b: n = 7; pattern c: n = 6; p > .05).

### EEG data acquisition and preprocessing

Continuous EEG data were recorded for 6–10 min in a resting-state with eyes closed during baseline (BASE), during the consolidation phase (CON), and after delayed retrieval (POST). During the experiment, all subjects were seated upright in a soundproofed and electromagnetic-shielded room. Participants were instructed in advance to avoid any movements, such as body movements, eye movements, and blinking. To ensure adequate vigilance of the subjects, their condition and ongoing EEGs were continuously monitored during the experiment. The EEG was recorded with the Neurowerk Amplifier (Headbox DB26, Neurowerk EEG software V13.19.2.73) from 19 active Ag/AgCl electrodes mounted in an elastic cap (MEDCAP). The system is based on the 10–20 electrode placement system. Two additional channels were used for recording electrooculograms. The ground electrode was located on the forehead (Fpz). The Cz electrode position served as an online reference channel. The electrode impedance was kept below 5 kΩ after careful preparation. The sampling rate was 512 Hz. EEG data were exported and converted to European Data Format (EDF) for preprocessing in EEGLAB (version 2021.1).

### EEG data preprocessing

Preprocessing and analysis of EEG data were performed using EEGLAB and its Darbeliai plugin (v2019.02.01.1, https://github.com/embar-/eeglab_darbeliai/wiki/0.%20EN) as well as in-house written MATLAB codes (The MathWorks Inc., Natick, MA). First, the EEG data were re-referenced to a common average reference in order to interpolate the Cz electrode. EEG data were filtered (0.3–45 Hz) using finite impulse response (FIR) filters. Channels were excluded and interpolated if (1) the channel received no signal for more than 5 s, (2) the signal frequency deviated by more than 4 standard deviations from the maximum accepted frequency, or (3) the signal had only a low correlation with the signal of the surrounding channels (*r* < 0.7). On average, 0.98 of the channels were removed and interpolated. The cleaned data were segmented into epochs of 4 s. The first 15 epochs were removed to allow participants to relax and enter a resting state. Finally, an Independent Component Analysis was computed and an automated identification and rejection of noise components using the ICLabel toolbox^51^ was performed followed by a visual inspection. The automated algorithm rejected an average of 1.75 Independent Components (ICs) and we manually removed an average of additional 1.72 ICs.

To calculate spectral power (μV^2^) the Fast Fourier Transformation was performed using the Darbeila plugin in EEGlab. The absolute power was calculated for the frequency range 1–45 Hz with a resolution of 0.1 Hz for each non-overlapping 2-s window of the first 30 artifact-free epochs. *Relative power* was obtained by normalizing absolute power in each frequency band (delta 1–4 Hz, theta 4–8 Hz, alpha 8–13 Hz, alpha1 8–11 Hz, alpha2 11–13 Hz, beta 13–30 Hz, gamma 30–45 Hz) with the total power within the whole frequency spectrum. The analysis of relative power was preferred to the analysis of absolute power because of its higher reliability.^52^

Due to the wide span of the frequency bands, relative power does not provide a particularly sensitive measure. Therefore, the PSD (10*log10 (μV2/ Hz)) for each 0.1 Hz step was additionally determined in the entire frequency spectrum and analyzed descriptively.

Individual alpha peak frequency (iAPF) was calculated by computationally determining the local peak in the PSD within the alpha frequency band for each resting-state session of both conditions and for each channel. If more than one local peak frequency was found, the peak with the highest power was selected. Since alpha frequency decreases with age,^53^ the lower bound of the bandwidth was exceeded. If iAPF was below 8 Hz, the PSD of the entire frequency spectrum was visually inspected by an experienced neurophysiologist and, if necessary, the iAPF was manually corrected. In addition, the power at iAPF (individual Alpha Peak Power, iAPP) was obtained. Two participants had to be excluded from the iAPF and iAPP analyses because no peak could be detected in the alpha frequency band (*N_AD_* = 13, *N_HC_* = 13).

### Statistical analysis

Statistical analysis was performed using SPSS (IBM SPSS Statistics; version 28, IBM Corp., Armonk, NY, USA). Because of the stimulation location in the parieto-occipital region and our particular interest in alpha oscillations generated mainly in posterior regions, our analyses focused on the occipital and parietal electrodes. To increase the signal-to-noise ratio for these analyses, the channels of the two brain regions of interest were averaged (parietal: P3, P4, Pz; occipital: O1, O2). To evaluate pathological changes, the relative power during the baseline resting-state was compared between groups (AD versus HC) and frequency bands (delta 1–4 Hz, theta 4–8 Hz, alpha 8–13 Hz, alpha1 8–11 Hz, alpha2 11–13 Hz, beta 13–30 Hz, gamma 30–45 Hz) using ANOVA. To evaluate the neurophysiological effect of rTMS on AD patients, relative power, iAPF, and iAPP were compared between groups (AD versus HC), conditions (STIM, stimulation versus SHAM, sham-stimulation) and session (BASE, baseline versus CON, consolidation versus POST, post-stimulation).

Performance in the memory task during the rTMS-EEG experiment was analyzed with respect to item memory and spatial context memory. Item memory was defined according to signal detection theory as d-prime (z[hit] – z[false alarm]), taking response bias into account. Spatial context memory was operationalized as positioning error, measured by the distance between the position of each stimulus that the participant indicated in the retrieval task and the position at which the stimulus was presented in the learning task. The distance was calculated as the root of the sum of the squared distances (measured in pixels) on the horizontal and vertical axes

$$(total\;distance=\sqrt{distance{\left(x\right)}^{2}+distance{\left(y\right)}^{2}}).$$

The lower the value, the better the spatial context memory. To examine pathologically decreased memory performance in the AD group on the one hand and the stimulation effect on memory performance on the other hand, item memory, and spatial context memory were compared between groups and stimulation conditions for both the initial and delayed recall. The interaction between delay (initial and delayed recall) and stimulation condition (STIM, SHAM) was used to assess a stimulation effect during the consolidation phase. Finally, correlation analyses were performed to test whether stimulation-induced changes in parieto-occipital alpha band activity are associated with memory performance.

## Results

### Behavioral results

Fishers exact test resulted in no significant difference for gender between groups (AD: 4 female, 10 male; HC: 7 female, 7 male; p = 0.44). For group differences in age, years of education and neuropsychological data see Table 2.

**Table 2.** Demographic and Neuropsychological Test Data and group comparison using bootstrapped independent t-test.

					*BCa 95% Confidence Interval*	
		HC	AD			
		*M (SD)*	*M (SD)*	*p*	Lower	Upper
	age (*SD*)	73.79 (7.47)	72.57 (7.29)	0.67^a^		
	years of education (*SD*)	14.71 (3.17)	15.57 (2.82)	0.59^a^		
cognitive domain	sub-domain					
	cognitive screening	29 (0.96)	24.64 (3.23)	0.01	2.93	6.03
verbal memory	Learning	22.71 (3.45)	11.64 (3.99)	<0.001	8.69	13.71
	Retrieval	8.21 (1.53)	2 (1.36)	<0.001	5.17	7.29
	Recognition	99.64 (1.34)	82.14 (10.14)	<0.001	12.81	22.85
visual memory	Retrieval	10.21 (1.37)	4.14 (2.93)	<0.001	4.11	7.79
visuospatial memory	positioning errors	15.07 (12.73	70.50 (40.64)	<0.001	−75.88	−33.49
	Learning	0.33 (0.16)	0.11 (0.18)	0.01	0.10	0.33
	Retrieval	−0.14 (0.53)	−2.86 (3.23)	0.02	1.13	4.49
	short term memory	6.79 (1.12)	6.43 (2.07)	0.57	−0.79	1.64
executive functions	cognitive flexibility	2.07 (0.48)	3.34 (2.03)	0.12	−2.61	−0.28
	working memory	6.00 (1.36)	4.79 (1.31)	0.02	0.19	2.16
attention	psychomotor speed	40.5 (9.96)	73.29 (48.37)	0.06	−60.81	−8.54
perceptual motor function	visuoconstructional ability	11 (0)	10.14 (1.41)	0.065	0.267	1.638
language	naming	14.86 (0.36)	12.29 (3.87)	0.09	0.73	4.71
	phonemic verbal fluency	15.14 (0.66)	12.64 (5.39)	0.13	−1.18	5.97
	semantic verbal fluency	23.21 (4.74)	15.71 (9.65)	0.03	1.66	13.46

Results are based on 1000 bootstrap samples. HC: healthy control group; AD: Alzheimer's disease group; BCa: bias corrected and accelerated bootstrapping.

^a^ Independent Samples T-test.

For spatial context memory, a significant main effect of *group* was found, with AD patients making more positioning errors than HC (*F*(1,26) = 17.80, *p* < 0.001, *ηp*^2^ = 0.41). A significant main effect of *delay* was found with more positioning errors in delayed than in initial recall (*F*(1,26) = 12.50, *p* = 0.002, *ηp*^2^ = 0.33). Furthermore, ANOVA revealed a significant *group x delay* interaction (*F*(1,26) = 6.63, *p* = 0.016, *ηp*^2^ = 0.20) and a significant *conditions x delay x group* interaction (*F*(1,26) = 4.43, *p* = 0.045, *ηp*^2^ = 0.15).

Post-hoc ANOVAs within groups revealed a significant main effect of *delay* only for AD (*F*(1,13 = 10.96, *p* = 0.006, *ηp*^2^ = 0.23). AD patients made more positioning errors in delayed than in initial recall, i.e., forgot spatial contextual information over time. The interaction *condition x delay* revealed no significant effect (*p* = 0.074), indicating that stimulation did not significantly influence the forgetting of spatial contextual information from initial to delayed recall in AD patients. There were no significant effects for HC.

For item memory, a significant main effect of *group* was evident across both stimulation conditions and both recall tasks, with decreased memory performance in AD compared with HC, *F*(1,26*)* = 10.86, *p* = 0.003, *ηp*^2^ = 0.30. No significant main effect of *condition* (stimulation versus sham stimulation) or *delay* (initial versus delayed recall), nor any significant interaction effects, were observed, all *p* > 0.05. For Descriptives see Table 3.

**Table 3.** Descriptives for item memory operationalized as d-prime [z(hit)-z(false alarm)] and spatial context memory operationalized as positioning errors, measured in pixels of distance.

	item memory			spatial context memory
	hit	false alarm	d-prime	positioning error
	*M (SD)*	*M (SD)*	*M (SD)*	*M (SD)*
AD				
initial recall				
stim	9.07 (1.64)	1.93 (3.22)	2.32 (1.26)	1068.20 (900.68)
sham	9.64 (0.75)	2.07 (3.47)	2.39 (1.23)	1090.91 (921.59)
delayed recall				
stim	9.36 (1.08)	1.50 (2.68)	2.47 (1.06)	1480.75 (1134.40)
sham	9.14 (1.29)	1.36 (2.13)	2.46 (0.96)	1165.56 (899.49)
HC				
initial recall				
stim	10 (0)	0.07 (0.27)	3.33 (0.16)	133.97(133.67)
sham	10 (0)	0 (0)	3.38 (0)	131.58 (218.48)
delayed recall				
stim	10 (0)	0.14 (0.36)	3.29 (0.21)	139.76 (229.71)
sham	9.93 (0.27)	0.21 (058)	3.23 (0.31)	202.48 (187.03)

AD: Alzheimer's disease; HC: healthy controls; stim: stimulation; sham: sham-stimulation; M: mean, SD: standard deviation.

### Relative power

**Figure 3.**
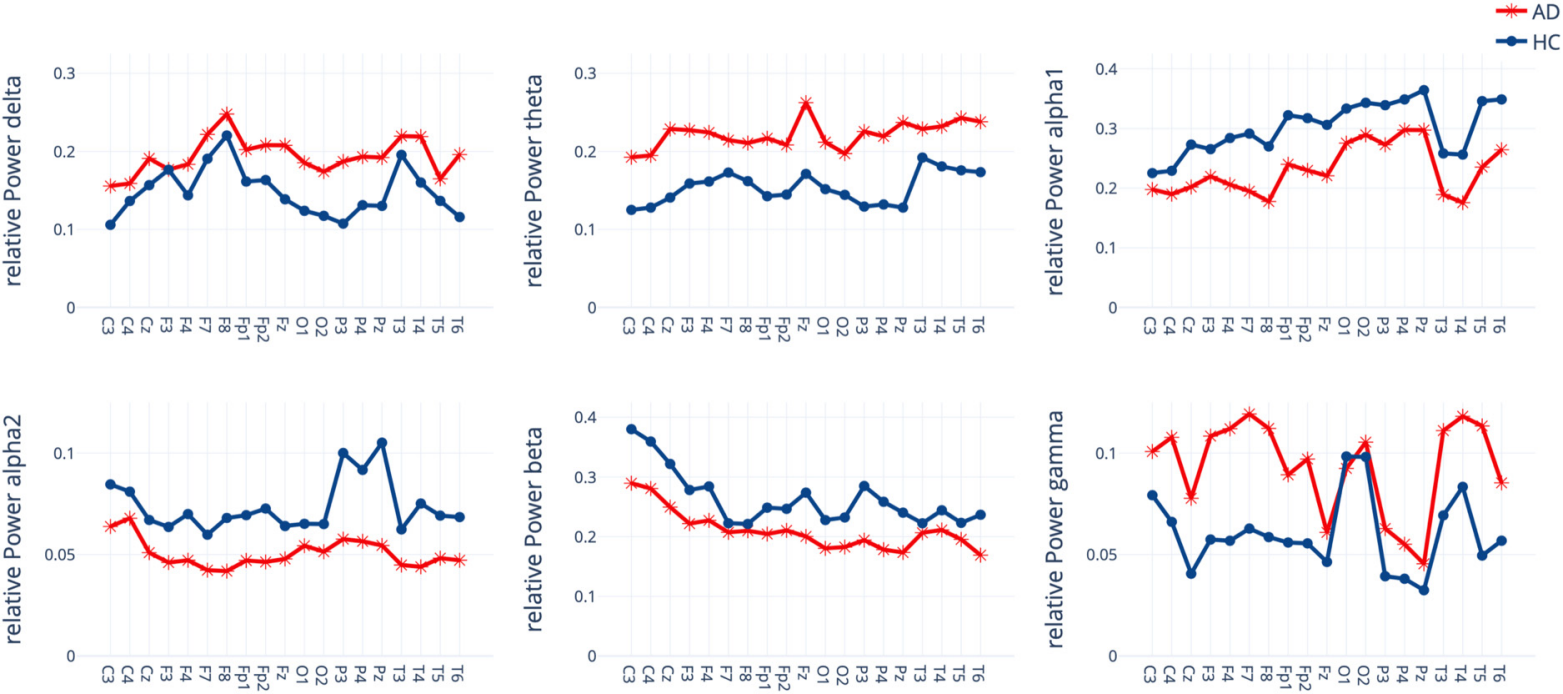
Relative Power for each channel within seven frequency bands for the Alzheimer's disease patients (AD) and healthy controls (HC) group.

For relative power during baseline, averaged over all channels, an ANOVA obtained a significant interaction effect *group* x *frequency band* (*F*(6156)= 3.90, *p* = 0.001, *ηp*^2^ = 0.13). Post-hoc t-tests showed significant group differences in theta (*p* = 0.01), alpha (*p* = 0.041) and alpha-2 (*p* = 0.028) bands with an increased relative theta and a decreased relative alpha power for AD compared to HC (see Figure 3).

**Figure 4.**
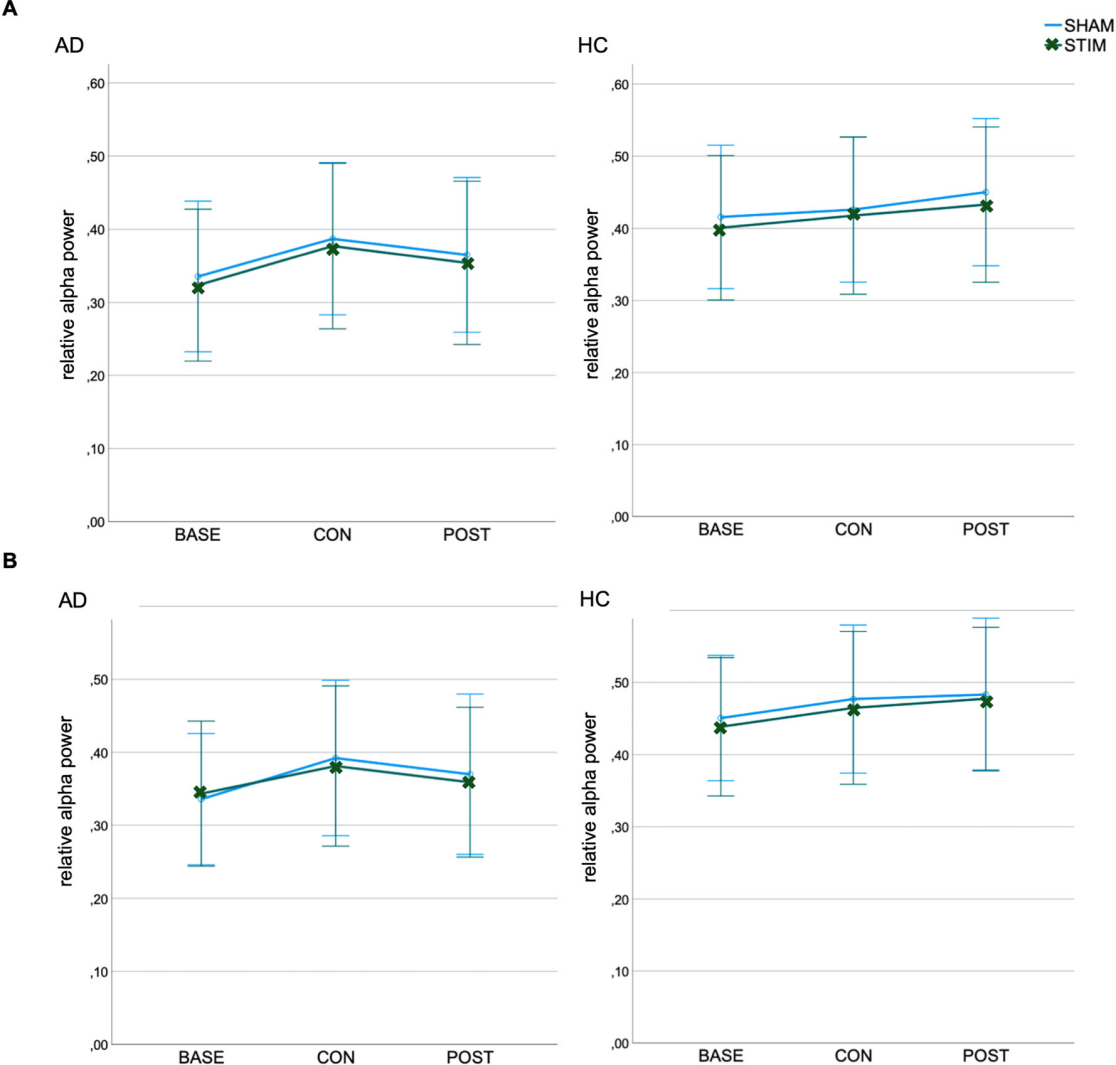
Relative Power in the alpha frequency band at (A) occipital and (B) parietal electrodes for Alzheimer's disease patients (AD) and healthy controls (HC). Bars represent the 95% confidence interval. BASE: baseline; CON: consolidation; POST: post-stimulation; STIM: stimulation; SHAM: sham-stimulation.

An ANOVA showed no effect of stimulation for relative Power within the alpha frequency band at either occipital or parietal electrodes. Regardless of the stimulation condition and group, relative Power differed between sessions (occipital: *F*(2,50) = 4.01, *p* = 0.024, *ηp*^2^ = 0.14; parietal: *F*(2,50) = 4.6, *p* = 0.015, *ηp*^2^ = 0.16) with an increase in relative alpha power from *baseline* to the second resting-state during the *consolidation* phase (occipital: *p* = 0.052, parietal: *p* = 0.028) and from *baseline* to *post* (occipital: *p* = 0.028, parietal: *p* = 0.035, see Figure 4). Differential analyses of alpha1 and alpha2 showed no effect of stimulation either.

### Power spectral density

**Figure 5.**
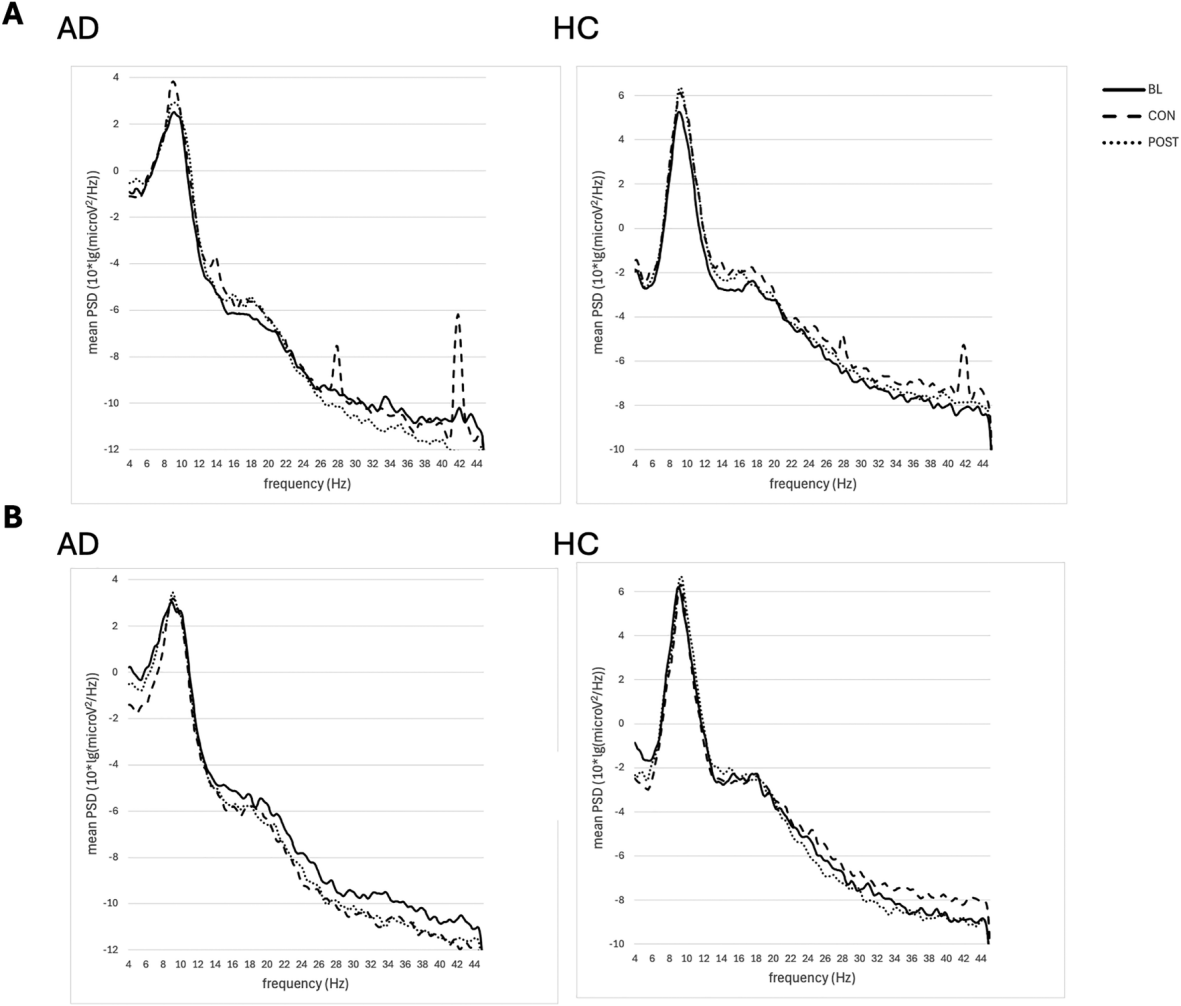
Occipital power spectral density (PSD) with a resolution of 0.1 Hz for Alzheimer's disease patients (AD) and healthy controls (HC) in the (A) stimulation and (B) sham stimulation condition. BASE: baseline; CON: consolidation; POST: post-stimulation.

Descriptively, at the mean peak frequency (*M_AD_* = 9.14, *SD_AD_* = 0.29, *M_HC_* = 9.08, *SD_HC_* = 0.22), an increase in the PSD was observed for HC and even more so for AD under active stimulation during the consolidation phase. This result was observed for all channels. Due to the stimulation site in the parieto-occipital region as well as our special interest in alpha oscillations, which are mainly generated in occipital regions, the following metrics and the figure are focused on data from occipital electrodes. Relative to baseline, PSD at the mean peak frequency increased by an average of 16.57% in HC under stimulation (*M_BASE_* = 5.25, *M_CON_* = 6.12) and an average of 1.94% under sham stimulation (*M_BASE_* = 6.19, *M_CON_* = 6.31). In AD, the PSD at the mean peak frequency increased by an average of 56.97% under stimulation (*M_BASE_* = 2.58, *M_CON_* = 4.05) and an average of 4.26% under sham stimulation (*M_BASE_* = 3.05, *M_CON_* = 3.18). In HC, the PSD increase maintained even after stimulation (*M_post_* = 6.35), whereas in AD PSD decreased immediately (*M_post_* = 2.66). See Figure 5.

An ANOVA on iAPF at occipital electrodes revealed significant interaction effects *stimulation* x *session* (*F*(2,48) = 3.90, Greenhouse-Geisser corrected *p* = 0.041, *ηp*^2^ = 0.15) and *group* x *session* (*F*(2,48) = 7.45, *p* = 0.002, *ηp*^2^ = 0.25). Further within-group analyses could not confirm a significant difference in baseline, consolidation or post between stimulation and sham stimulation neither for AD nor HC. An ANOVA on iAPF at parietal electrodes revealed no significant main or interaction effects.

**Figure 6.**
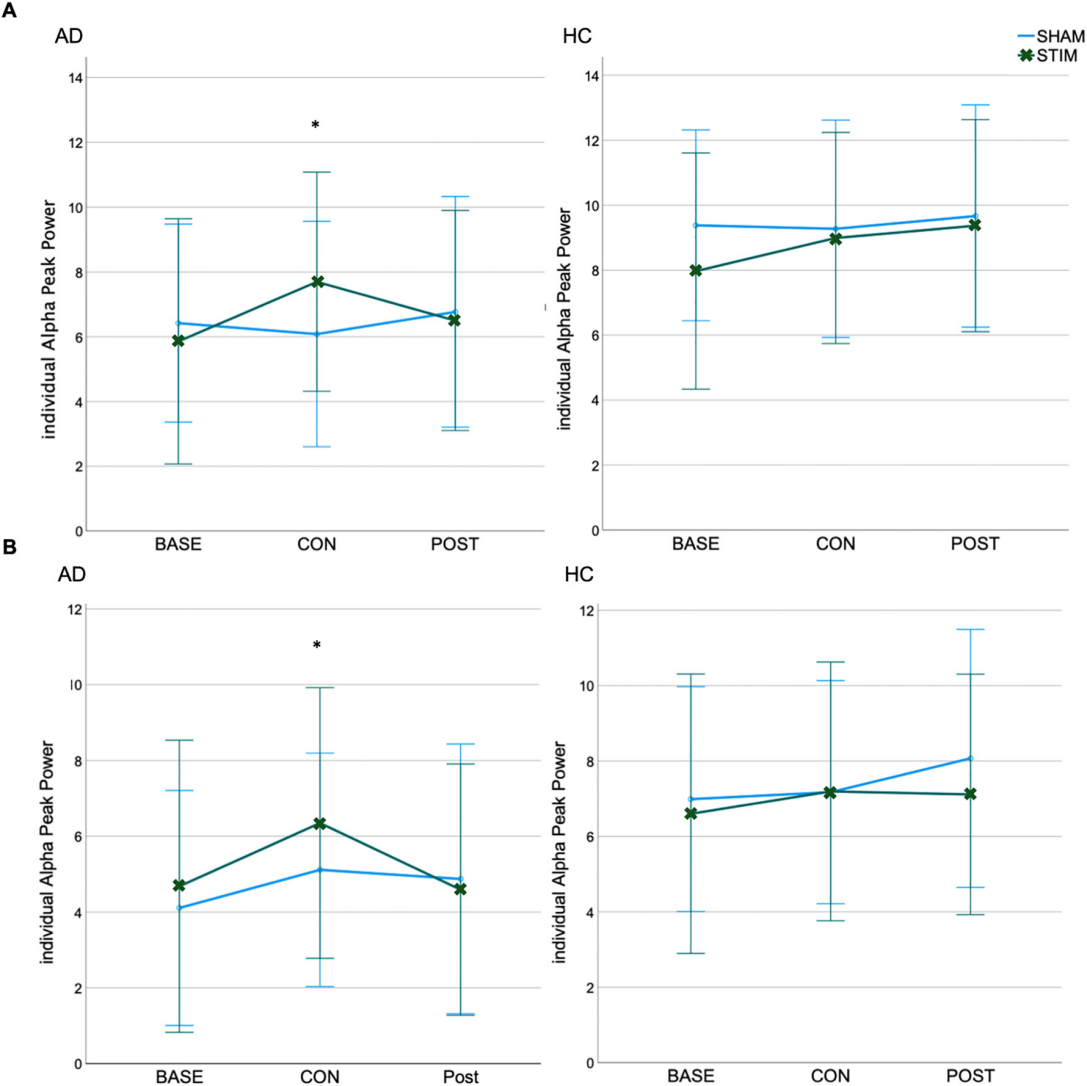
Individual Alpha Peak Power (iAPP) at (A) occipital and (B) parietal electrodes for Alzheimer's disease patients (AD) and healthy controls (HC) group. *p < 0.05; Bars represent the 95% confidence interval. BASE: baseline; CON: consolidation; POST: post-stimulation; STIM: stimulation; SHAM: sham-stimulation.

Within-group analyses on iAPP revealed significantly higher peaks under stimulation (consolidation phase) compared to sham stimulation in the AD group, both occipitally (t(12) = 2.25, p = 0.046, 95%CI [0.24, 2.93], Cohens’ d = 0.65) and parietally (t(12) = 2.24, p = 0.047, 95%CI[0.15, 2.27], Cohens’ d = 0.65) by 26.64% and 24.26%, respectively. There were no significant differences in iAPP during *baseline* or *post*. No significant simulation-related changes in iAPP were detected in the HC group. See Figure 6.

IAPP at occipital and parietal electrodes during the consolidation phase is positively correlated with item memory in the delayed recall only under sham stimulation (sham: occipital: *r* = 0.57, *p* = 0.004, *95%CI*[0.20, 0.80]; parietal: *r* = 0.48, *p* = 0.017, *95%CI*[0.19,0.74]; Stim: occipital: *r* = 0.35, *p* = 0.093, *95%CI*[0.06, 0.59]; parietal: *r* = 0.31, *p* = 0.141, *95%CI*[-0.5, 0.58]). Within-groups analyses show no significant correlations (all p > 0.05).

## Discussion

The aim of the present study was to investigate the underlying neurophysiological mechanisms of high-frequency low-intensity rTMS over parieto-occipital brain regions and its effect on memory performance in AD patients and cognitively healthy seniors. We were able to demonstrate that during rTMS alpha power increased compared to sham. In AD patients, the stimulation protocol used, appeared to be beneficial with respect to the pathologically altered alpha band. Under stimulation, iAPP in AD patients increased descriptively by about 25 percent relative to sham, suggesting a therapeutic effect. Interestingly, in age-matched healthy seniors no stimulation effect on iAPP was evident. This difference in responsiveness between AD patients and age-matched healthy seniors could be explained by the difference in their baseline neuronal activity. According to the principle of state dependence, different brain states imply a different susceptibility to the stimulation effect.^54^ Accordingly, the results of the current study show a stronger stimulation effect on the alpha band in AD patients with pathologically lowered alpha at baseline than in healthy control subjects with a comparatively high alpha at baseline. A supplementary correlation analysis further confirmed the state dependency principle. The lower the individual alpha power in baseline, the higher the increase of alpha from baseline to consolidation (Δ iAPP consolidation – baseline) under stimulation (*r* = -0.473, *p* = 0.015, *95%CI* [−0.665, −0.151]). A number of studies have systematically investigated this effect on NIBS by inducing different brain states in terms of different baseline alpha levels by two different conditions: eyes open and eyes closed.^30,55^ Occipital alpha reflects the wakeful resting state, marked by reduced sensitivity to external stimuli, including blocking visual input, and an increased focus on internal representations. Accordingly, alpha oscillations synchronize when the eyes are closed and desynchronize when the eyes are open.^55^ The two conditions – *eyes open* and *eyes closed* represent different brain states, with eyes closed associated with higher alpha power and eyes open with lower alpha power.^56^ Studies have shown that stimulation with alpha-tACS over parieto-occipital brain regions had a significantly stronger effect on the alpha band when participants kept their eyes open than when they kept their eyes closed. Conclusively, studies demonstrate a stronger response to the stimulation of the alpha band at lower baseline alpha levels, which is in line with the results of the current study.^30,55^

There are two main hypotheses regarding the underlying mechanisms of frequency-guided noninvasive brain stimulation (i.e., rhythmic TMS and tACS).^57^ In many brain regions, neural circuits function like oscillators: neural signals travel from neuron to neuron, and the speed at which these signals propagate through the circuit determines its resonance frequency—that is, the frequency to which the network responds most effectively. The first hypothesis states that the stimulation effect is mainly due to the entrainment of those resonance frequencies by the stimulation frequency. The stimulation frequency serves as a kind of pace generator. The stimulation effect is caused by the synchronization of the natural oscillations to an external source – the stimulation frequency.^29^ Accordingly, the power increase is expected to be centered at the stimulation frequency, i.e., 14 Hz in the current study, and not at the intrinsic eigenfrequency for the alpha band.

In contrast, the second hypothesis – the *plasticity hypothesis –* states that the stimulation effect is due to Spike-Timing-Dependent Plasticity (STDP). STDP describes a principle according to which the strength of a synaptic connection changes depending on the temporal sequence of neural signals. If the presynaptic neuron fires shortly before the postsynaptic neuron, the connection is strengthened (long-term potentiation; LTP). If the opposite happens, i.e., if the postsynaptic neuron is activated first, the connection is weakened (long-term depression; LTD).^58^ Zaehle et al.^59^ assume that brain stimulation applied at a frequency that matches the resonance frequency of a network, the synapses involved can be strengthened by STDP-like processes. These changes can continue even after stimulation and lead to increased activity in the affected network. According to this assumption, no shift of the iAPF would occur, but power enhancement would be observed at the intrinsic eigenfrequency for the alpha band (iAPP).

Our results show a significant increase in PSD at the stimulation frequency (14 Hz) and its harmonics (28 Hz, 42 Hz; Figure 5). However, AD patients show an equally significant increase in PSD at the mean alpha peak frequency at around 9 Hz. Thus, stimulation did not cause an alignment in spontaneous alpha frequency, which is also confirmed by our results on iAPF, according to which no stimulation-induced shift of the iAPF was found in either AD or HC. Moreover, the observed effects at the stimulation frequency (14 Hz) could be artificially generated rather than endogenous brain oscillations. Even if, according to the entrainment hypothesis, an oscillatory response of the brain was elicited by stimulation at this frequency, it could not be distinguished from an artificially generated signal. Conversely, this also means that the observed power enhancement at the iAPF (iAPP) most likely did not originate from an artificial signal. Hence, our results support the *plasticity hypothesis* and thus confirm and extend the systematic investigation by Vossen et al.^57^ as well as numerous studies on long-lasting after-effects of stimulation, which could not be explained by entrainment as the underlying mechanism.^31,55,59^ However, since our results only showed effects during stimulation but no effect lasting after stimulation, it is likely that the applied single rTMS session induced short-term facilitation of synaptic activity but was not sufficient enough to induce long-term potentiation. Repeated stimulation sessions might be required to induce more persistent plastic changes.

Regarding our secondary outcome variables, item and spatial context memory, no stimulation effect was found either for AD patients or for healthy seniors. In the case of healthy seniors, a ceiling effect appears to be the most probable explanation, given that the vast majority of participants demonstrated an accurate recollection of all items and the spatial context in both conditions, both prior to and following the delay. However, this explanation is not applicable for AD patients. The finding of no significant difference in memory performance between the stimulation and sham conditions challenges the prevailing hypothesis that stimulation induced alpha power enhancement leads to improved memory performance.

One possible explanation could be the differential influence of increased alpha power during different memory processes. During encoding and retrieval, a decrease in alpha power—often referred to as alpha desynchronization—is commonly observed in task-relevant brain regions, such as parietal areas.^60^ This desynchronization reflects increased cortical excitability and has been associated with enhanced information processing and successful memory formation.^60–63^ A recent study by Martín-Buro et al.^64^ even found that alpha power gradually decreased from a cued recall to an item recognition task and from an item recognition task to a recall of associated contextual information, representing a gradual accumulation of memory strength.

In contrast, during maintenance or consolidation phases, where external input is minimal and internal representations are prioritized, an increase in alpha power may be beneficial. This increase likely reflects functional inhibition of task-irrelevant sensory areas, facilitating focused internal processing.^65^ Supporting this view, Khader et al.^66^ found that higher posterior alpha power during the maintenance/consolidation interval predicted better subsequent long-term memory performance, suggesting that alpha synchronization can serve a supportive role when memory traces are stabilized in the absence of external stimulation. Thus, the cognitive relevance of alpha activity depends on the temporal dynamics of the memory process: desynchronization during encoding and retrieval, but synchronization during maintenance or consolidation.

Artificially inducing synchronization of alpha oscillations/ increasing alpha power in the parietal brain areas during encoding and retrieval may interfere with the underlying neurophysiological processes and impair memory. This hypothesis is consistent with the findings of Gonzalez-Rosa et al. (2015). They observed that a stimulation-induced increase in alpha power resulted in a longer reaction time (RT) in a visual detection task, and not, as the authors expected, a shortened RT and thus better performance. A positive effect of stimulation during consolidation and a negative effect of stimulation during encoding and retrieval could have counterbalanced each other. This could explain why, compared to sham stimulation, we did not observe a significant improvement in memory performance but rather a nonsignificant trend toward greater forgetting from initial to delayed retrieval in AD patients under stimulation. However, the present study cannot conclusively clarify at which stage of memory formation stimulation of the alpha frequency band has positive effects on memory performance. Further research is needed using a standardized stimulation protocol to stimulate different memory processes at different times.

### Limitations

Several limitations of this study should be acknowledged. First, the sample size was relatively small, largely due to the high economic costs of data collection. Participants were required to attend three clinic visits, each lasting at least 90 min. Despite this, participant selection was highly rigorous: the AD group included only patients with a biomarker-confirmed diagnosis of AD, and control and patient groups were matched for age, gender, and education. Second, although the stimulation protocol was designed to be individualized, it also required a certain degree of standardization. To achieve this balance, three different stimulation patterns were tested to identify the most effective one for each participant. This procedure, however, resulted in variability in the total pulse dose between participants. Future studies should aim to optimize individualization procedures while maintaining consistent stimulation parameters across subjects. Third, stimulation was not neuronavigated. Instead, we deliberately applied a broader stimulation approach, as the device generates very low-intensity magnetic fields that affect cortical networks rather than single, well-defined regions. While this approach was intended to target large-scale posterior networks, it limits the spatial precision of the stimulation. Fourth, the stimulation protocol involved only a single rTMS session. To investigate potential long-lasting after-effects and cumulative plastic changes, future studies should employ repeated stimulation sessions over extended periods. Finally, the study employed a single-blind design. Developing the stimulation device further to allow for fully double-blinded experimental conditions would strengthen the methodological rigor of future investigations.

### Conclusion

Conclusively, the present work has extended the existing knowledge on the underlying mechanisms of NIBS. Our results show that rTMS above parieto-occipital brain areas at alpha frequency level can modulate pathological brain activity in AD patients even at low intensities. Future research is needed to investigate the sustained therapeutic effect when stimulation is applied regularly over several weeks or months.^10,37^ To achieve a sufficient sample size for this purpose, a multi-center study is recommended.
